# Quantitative Analysis of the *Arabidopsis* Leaf Secretory Proteome via TMT-Based Mass Spectrometry

**DOI:** 10.21769/BioProtoc.5508

**Published:** 2025-11-20

**Authors:** Sakharam Waghmare, Lingfeng Xia, Suzanne McGill, Richard Burchmore, Rucha Karnik

**Affiliations:** 1 Plant Science Group, School of Molecular Biosciences, College of Medical, Veterinary and Life Sciences, University of Glasgow, Bower Building, University Avenue, Glasgow, UK; 2 School of Infection & Immunity, College of Medical, Veterinary and Life Sciences, University of Glasgow, Sir Graeme Davies Building, Gilmorehill Campus, University Place, Glasgow, UK

**Keywords:** Arabidopsis, Apoplast flush collection, Tandem mass tag (TMT), Liquid chromatography–mass spectrometry (LC-MS), Filter-aided sample preparation (FASP), Proteome Discoverer, MSPepSearch, Spectral library, Quantitative plant proteomics

## Abstract

In plants, the apoplast contains a diverse set of proteins that underpin mechanisms for maintaining cell homeostasis, cell wall remodeling, cell signaling, and pathogen defense. Apoplast protein composition is highly regulated, primarily through the control of secretory traffic in response to endogenous and environmental factors. Dynamic changes in apoplast proteome facilitate plant survival in a changing climate. Even so, the apoplast proteome profiles in plants remain poorly characterized due to technological limitations. Recent progress in quantitative proteomics has significantly advanced the resolution of proteomic profiling in mammalian systems and has the potential for application in plant systems. In this protocol, we provide a detailed and efficient protocol for tandem mass tag (TMT)-based quantitative analysis of *Arabidopsis thaliana* secretory proteome to resolve dynamic changes in leaf apoplast proteome profiles. The protocol employs apoplast flush collection followed by protein cleaning using filter-aided sample preparation (FASP), protein digestion, TMT-labeling of peptides, and mass spectrometry (MS) analysis. Subsequent data analysis for peptide detection and quantification uses Proteome Discoverer software (PD) 3.0. Additionally, we have incorporated in silico–generated spectral libraries using PD 3.0, which enables rapid and efficient analysis of proteomic data. Our optimized protocol offers a robust framework for quantitative secretory proteomic analysis in plants, with potential applications in functional proteomics and the study of trafficking systems that impact plant growth, survival, and health.

Key features

• Rapid and high-purity collection of *Arabidopsis thaliana* leaf apoplast flush.

• Use of filter-aided sample preparation (FASP) for protein cleaning to obtain high-quality data.

• Use of in-house-generated theoretical spectral libraries for efficient and rapid analysis of MS data.

## Graphical overview


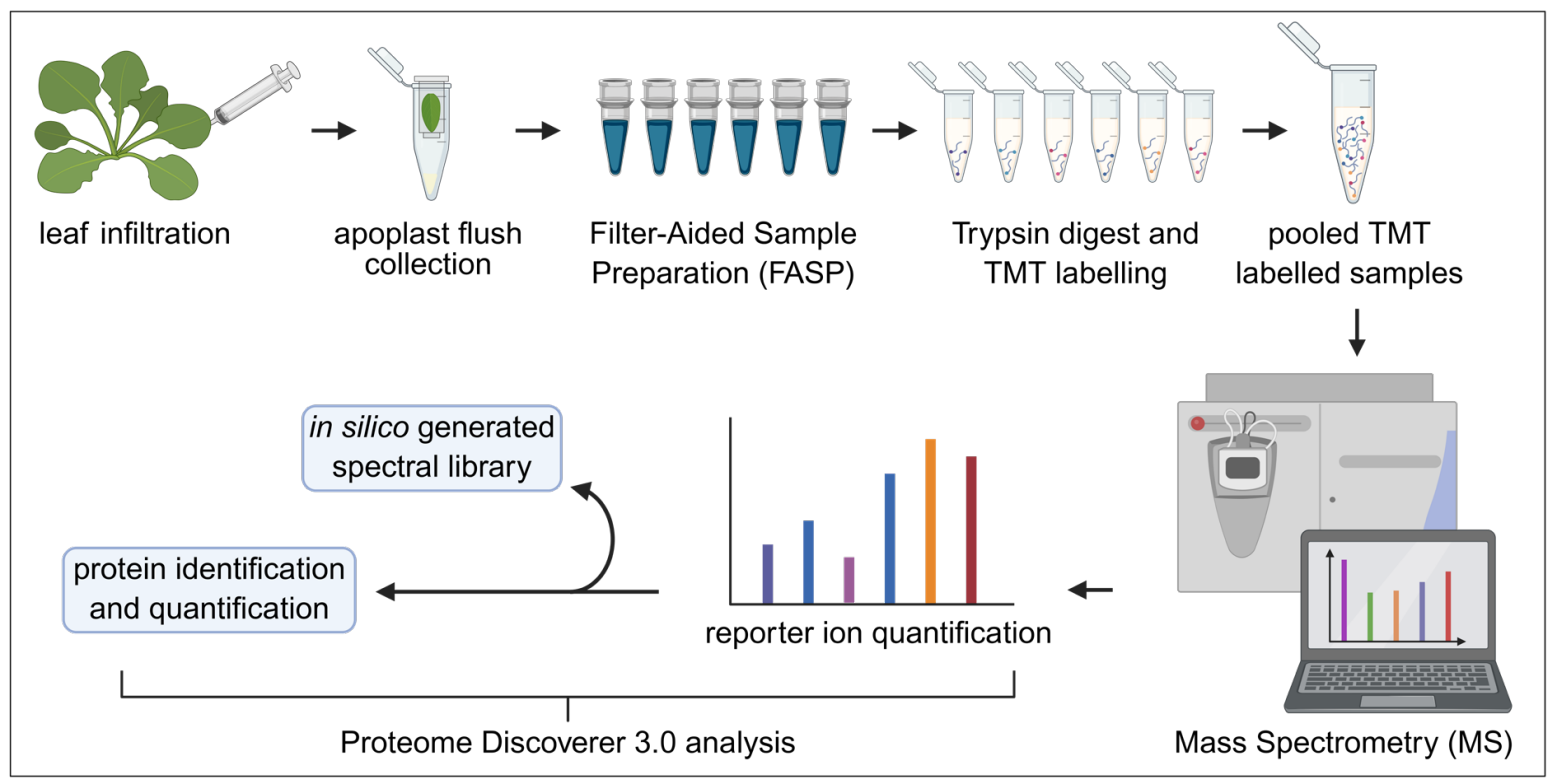


**Tandem mass tag (TMT)-mass spectrometry (MS) analysis of *Arabidopsis* secretory proteome**

## Background

Plants, as sessile organisms, are constantly exposed to multifactorial abiotic and biotic stresses in both natural and agricultural environments. The apoplast serves as a critical interface between the plant cell and its environment, containing a dynamic array of secretory proteins that contribute to cell wall remodeling, cell signaling, abiotic stress, and host-defense mechanisms [1–3]. In order to survive in a challenging environment, plants must dynamically regulate apoplast protein composition, relying on a combination of constitutive and environment-sensitive secretory pathways active at the cell membrane [4–8]. To understand plant adaptations to climate change and elucidate secretory mechanisms in plant cells, a detailed understanding of dynamic changes in the plant secretory proteome profile is crucial. The knowledge gained will feed future strategies and interventions to enhance plant immunity and resilience to climate change, thereby improving agricultural production.

Plant proteomics research is gaining momentum for the high-throughput profiling of secretory proteins [1]. This process typically involves liquid chromatography (LC) coupled to tandem mass spectrometry (MS/MS) and bioinformatics technology. A label-free quantification technique is commonly utilized for apoplast profiling. Although the label-free technique offers benefits such as cost-effectiveness, higher proteome coverage, and flexibility, its major disadvantage is lower accuracy, sensitivity, and limited multiplexing [9–11]. In contrast, label-based quantification, such as tandem mass tag (TMT), offers high precision and accuracy, facilitating multiplexing up to 35 samples. TMT kits are commercially available in 6-, 10-, 11-, 16-, 18-, and 35-plex formats, allowing user selection based on their specific experimental needs [12–14] and enabling simultaneous quantitative analysis of samples under different conditions and biological replicates in a single run [15–17]. TMT-labeled spectral libraries, collections of reference spectra representing peptides, are commonly used in other eukaryotes and prokaryotes, yet they remain rare in plant proteomics. We outline here a protocol that harnesses this TMT labeling strategy for quantitative plant proteome profiling.

Plant secretory proteomic analysis is a complex multi-step experimental workflow that relies on efficient sample collection, combined with biochemistry, proteomics, and bioinformatics in a precise manner, with each step posing distinct challenges. Common challenges include cytoplasmic protein and nonprotein contaminants, which can affect sample processing in subsequent stages as well as proteomic analysis [18]. Highly abundant cytoplasmic contaminants, such as RUBISCO, hinder MS analysis by overshadowing the low-abundant secretory proteins. During apoplast flush collection, cytoplasmic leakage due to cell damage cannot be completely avoided. Therefore, it is important that sample preparation is optimized to minimize cytosolic contamination and ensure uniformity in sample preparation for reliable data collection.

There are several advanced tools available for MS data analysis, but their use in plants is limited. Compared to conventional sequence searching, spectral library searching is faster and provides higher specificity and sensitivity [19]. While unlabeled spectral libraries for *Arabidopsis*’s proteome are available [20], the TMT-labeled spectral libraries are lacking. Proteome Discoverer 3.0/3.1 (PD 3.0/3.1) allows the prediction of TMT-labeled spectral libraries, effectively addressing this gap. We successfully utilized TMT-based (TMT 6-plex, 11-plex, and 18-plex) quantitative mass spectrometry to characterize the *Arabidopsis* secretory proteomic profiles from the leaf apoplast [21]. In doing so, we were able to demonstrate for the first time that TMT–MS analysis, coupled with analysis using predicted spectral libraries via Proteome Discoverer^TM^ software, achieves enhanced peptide detection. The strategy has enabled high-resolution proteome analysis to resolve the mechanism of the SNARE-assisted secretory pathways during bacterial pathogenesis in plants.

**Figure 1.**
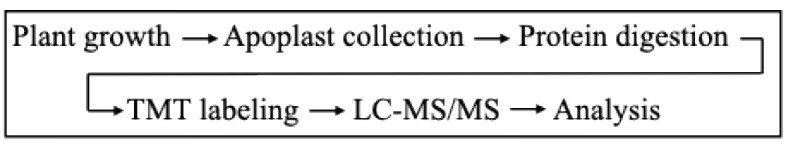
Workflow diagram

This protocol focuses on the analysis of the *Arabidopsis* secretory proteome using the TMT 6-plex approach. The protocol consists of the following major steps: apoplast flush collection, protein cleaning, digestion, TMT labeling, MS analysis, and subsequent data analysis using PD3.0 (see Figure 1). The primary benefits of this optimized protocol include (1) rapid collection of apoplast flush in bulk amount with minimal cytoplasmic protein contamination, (2) filter-aided sample preparation (FASP) for protein cleaning, consistently generating high-quality data, (3) the use of spectral libraries for efficient and rapid analysis of MS data, and (4) scalable method up to 10-, 11-, 16-, and 18-plex TMT labeling and analysis. For further details, including the results and analysis process, please refer to Waghmare et al. [21].

## Materials and reagents

**CAUTION:** General laboratory safety precautions should be taken while working with flammable, corrosive, and toxic chemicals, including the use of a fume hood and appropriate personal protective equipment, while adhering to regulations that follow standard health and safety risk assessments.

*Note: Store reagents as per the manufacturer’s instructions and check use-by date.*

**Biological materials**

1. *Arabidopsis thaliana* Columbia-0 ecotype (Col-0, ABRC stock CS60000) and transgenic lines; store seeds at 4 °C in dry conditions for maximum viability

**Reagents**

1. Sodium dodecyl sulphate (SDS) (Sigma-Aldrich, catalog number: L4509)

2. Tris base (Sigma-Aldrich, catalog number: T6066)

3. Dithiothreitol (DTT) (Sigma-Aldrich, catalog number: D9163)

4. Urea (Sigma-Aldrich, catalog number: U5128)

5. HCl (Fisher Chemical, catalog number: 10294190)

6. Iodoacetamide (IAA) (Sigma-Aldrich, catalog number: 1149)

7. Ammonium bicarbonate (Sigma-Aldrich, catalog number: A6141)

10. Trypsin (Promega, catalog number: V5111)

12. Acetonitrile (Rathburn LCMS-Grade, catalog number: RH1016LCMS)

13. Triethylammonium bicarbonate (TEAB) (Sigma-Aldrich, catalog number: T7408)

14. MgCl_2_ (Sigma-Aldrich, catalog number: M8266)

15. Laemmli buffer (Bio-Rad, catalog number: 1610747)

16. Gel stain (Neo Biotech, catalog number: NB-45-00078)

17. Bradford protein assay dye reagent concentrate (Bio-Rad, catalog number: 5000006)

18. Acetonitrile (HPLC-grade) (Rathburn LCMS-Grade, catalog number: RH1016LCMS)

19. Formic acid (Thermo Fisher Scientific, catalog number: A117)

20. TFA (Thermo Fisher Scientific, catalog number: 85183)

21. TMT Isobaric Mass Tagging Kit, 6-plex, 10-plex, 16-plex (Thermo Fisher Scientific)

22. Hydroxylamine (Thermo Fisher Scientific, catalog number: 90115)

23. Distilled water

**Solutions**

1. Infiltration buffer (see Recipes)

2. SDT-lysis buffer (see Recipes)

3. 0.1 M Tris/HCl (see Recipes)

4. UA (see Recipes)

5. IAA (see Recipes)

6. Trypsin (see Recipes)

7. 10% acetonitrile (see Recipes)

8. 50 mM ammonium bicarbonate (see Recipes)

9. 100 mM TEAB (see recipes)

10. 5% hydroxylamine (quenching solution) (see Recipes)

11. Reverse-phase UHPLC solvent A (see Recipes)

12. Reverse-phase UHPLC solvent B (see Recipes)

13. Sample resuspension buffer (see Recipes)

**Recipes**

**1. Infiltration buffer**

**Table:** 

Reagent	Final concentration	Quantity for 100 mL
1 M MgCl_2_	10 mM	1 mL
Autoclaved MilliQ water		99 mL

*Note: Store at 4 °C.*

**2. SDT-lysis buffer**

**Table:** 

Reagent	Final concentration	Quantity for 100 mL
10% SDS	4% (w/v)	40 mL
1 M Tris/HCl (pH 7.6)	100 mM	10 mL
1 M DTT	100 mM	10 mL
Autoclaved MilliQ water		to 100 mL

*Note: Store at room temperature.*

**Caution:** Wear a mask and gloves when handling SDS powder to avoid dust inhalation.

**3. 0.1 M Tris/HCl**

**Table:** 

Reagent	Final concentration	Quantity for 1 L
1 M Tris/HCl (pH 8.5)	0.1 M	100 mL
Autoclaved MilliQ water		900 mL

*Note: Store at 4 °C.*

**4. UA**

**Table:** 

Reagent	Final concentration	Quantity for 10 mL
8.8 M Urea	8 M	9
1 M Tris/HCl (pH 8.5)	100 mM	1

*Note: Prepare freshly and use within 1 day. 1 mL per sample.*

**5. IAA**

**Table:** 

Reagent	Final concentration	Quantity for 1 mL
0.5 M iodoacetamide	50 mM	100 μL
UA		900 μL

*Note: Store at 4 °C. Prepare freshly and use within 1 day. 0.1 mL per sample.*

**6. Trypsin**

**Table:** 

Reagent	Final concentration	Quantity for 100 μL
1 μg/μL trypsin	0.05 μg/μL	5 μL
1 M ammonium bicarbonate (pH 8.3)	50 mM	95 μL

*Note: Store at -20 °C. The enzyme-to-protein ratio should be 1:100. For 25 μg of protein, use 0.25 μg of trypsin.*

**7. 10% acetonitrile**

**Table:** 

Reagent	Final concentration	Quantity for 1 L
Acetonitrile	10%	100 mL
Autoclaved MilliQ water		900 mL

*Note: Store at room temperature.*

**8. 50 mM ammonium bicarbonate**

**Table:** 

Reagent	Final concentration	Quantity for 100 mL
1 M ammonium bicarbonate (pH 8.3)	50 mM	5 mL
Autoclaved MilliQ water		95 mL

*Note: Prepare 0.5 mL per 1 sample. Store at -20 °C.*

**9. 100 mM TEAB**

**Table:** 

Reagent	Final concentration	Quantity for 1 L
1 M triethylammonium bicarbonate	100 mM	100 mL
Autoclaved MilliQ water		900 mL

*Note: Store at -20 °C.*

**10. 5% hydroxylamine (quenching solution)**

**Table:** 

Reagent	Final concentration	Quantity for 1 mL
50% hydroxylamine	5%	0.1 mL
Autoclaved MilliQ water		0.9 mL

*Note: Store at room temperature.*

**Caution:** Wear a mask and gloves when handling. Avoid release to the environment.

**11. Reverse-phase UHPLC solvent A**

**Table:** 

Reagent	Final concentration	Quantity for 100 mL
Formic acid	0.01%	10 μL
Autoclaved MilliQ water		to 100 mL

*Note: Store at room temperature.*

**12. Reverse-phase UHPLC solvent B**

**Table:** 

Reagent	Final concentration	Quantity for 100 mL
Formic acid	0.08%	80 μL
Acetonitrile	80%	80 mL
Autoclaved MilliQ water	20%	to 100 mL

*Note: Store at room temperature.*

**13. Sample resuspension buffer**

**Table:** 

Reagent	Concentration	Quantity for 10 mL
Formic acid	0.50%	50 μL
Acetonitrile	1%	100 μL
Autoclaved MilliQ water		to 10 mL

*Note: Store at room temperature.*

**Laboratory supplies**

1. Soil mix

2. Plant pots (with holes)

3. Plant trays without drain holes

4. Plant growth room/chamber

5. Forceps, sticky tapes, markers

6. Wash bottles for watering (500 mL)

7. Falcon tubes 15 mL (Corning, catalog number: 430790) and 50 mL (Corning, catalog number: 430828)

8. Microfuge tubes 1.5 mL (Sarstedt, catalog number: 72.690.001) and 0.5 mL (Sarstedt, catalog number: 72.699)

9. Syringes 1 mL (Thermo Fisher Scientific, catalog number: 15489199) and 10 mL (Thermo Fisher Scientific, catalog number: 15205093)

10. Disposable gloves, forceps, paper towels, blades

11. Micropipettes (100 μL, 1 mL)

12. Protein ladder (Thermo Fisher Scientific, catalog number: 26619)

13. NuPAGE 4%–12% bis-tris gel (Invitrogen, catalog numbers: NP0321BOX and NP0322BOX)

14. Filter unit (Millipore, model: Ultracel YM-30)

15. 96-well plate (Greiner Bio-One, catalog number: 651101)

16. C18 trap column (0.3 × 5 mm) PepMap Neo C18 5 μm, 300 μm × 5 mm 17400 (Thermo Scientific)

17. Acclaim Pepmap RSLC C18 reversed phase column (50 cm × 75 μm nano viper, particle size 2 μm, pore size 100 Å) (Thermo Scientific, model: SN20579764)

## Equipment

1. Vortex (Scientific Industries, model: Vortex-Genie^®^ 2 mixer)

2. Autoclave (Rodwell, model: ST2228V)

3. Refrigerated centrifuge (fixed angle rotor) (Eppendorf, model: 5424R, fixed-angle rotor)

4. Powerpack

5. SDS-PAGE gel apparatus (Life Technology, catalog number: A25977)

6. Wet chamber/heat block set to 37 °C

7. Liquid chromatography (LC): Dionex Ultimate 3000 RSLC nanoflow system (Dionex, Camberley, UK)

8. Electrospray ionization (ESI) mass spectrometry: Orbitrap Elite/fusion (Thermo Fisher Scientific, UK)

## Software and datasets

1. Database: Arabidopsis taxonomy from the NCBI database

2. Software: Proteome Discoverer 3.0 software (Thermo Fisher Scientific, UK)

## Procedure

**A. Plant propagation**

*Note: The use of actively growing* Arabidopsis *plant tissue is recommended. Optimal plant age may vary based on transgenic lines and/or treatments. In this protocol, leaves excised from 3–4-week-old* Arabidopsis *plants were used.*

1. Sow *Arabidopsis* seeds (approximately 50) in a pot filled with soil (we used 6.5 cm diameter plastic pots). Transfer the pots to a plastic tray and cover the tray to maintain humidity. Incubate at 4 °C in the dark to promote vernalization for 2–3 days to improve the germination rate and uniformity.

2. Transfer the pots to the plant growth facility for approximately 8–10 days to allow plant growth at 22 °C with 8/16 h light/dark cycle, 150 μmol·m^-2^·s^-1^ white light, and a relative humidity of 55%–60% as a standard condition for *Arabidopsis* propagation.

*Note: Other growth conditions may be used as relevant for the experiment.*

3. Carefully transplant individual seedlings into plastic pots with holes on the bottom to facilitate watering from below (we used square 6.5 cm diameter plastic pots). Place the pots on a tray, cover, and move to the growth chamber for plant propagation.

*Note: One MS run typically requires three biological replicates. The number of plants will depend on the number of leaves on the rosette and their size. We recommend growing at least 2–3 extra plants to account for any leaf damage during sample preparation.*

4. Once the seedlings are established (approximately seven days after planting), uncover the tray and grow the plants for two more weeks.

5. Plants are ready for apoplast flush collection when they have a robust rosette of 10–12 leaves (approximately 20 days after planting).

**B. Apoplast isolation**

*Note: Samples prepared using undamaged leaves of similar size/age across all replicates/treatments are recommended to obtain consistent results. Larger leaves could be sliced into strips.*

**Figure 2.**
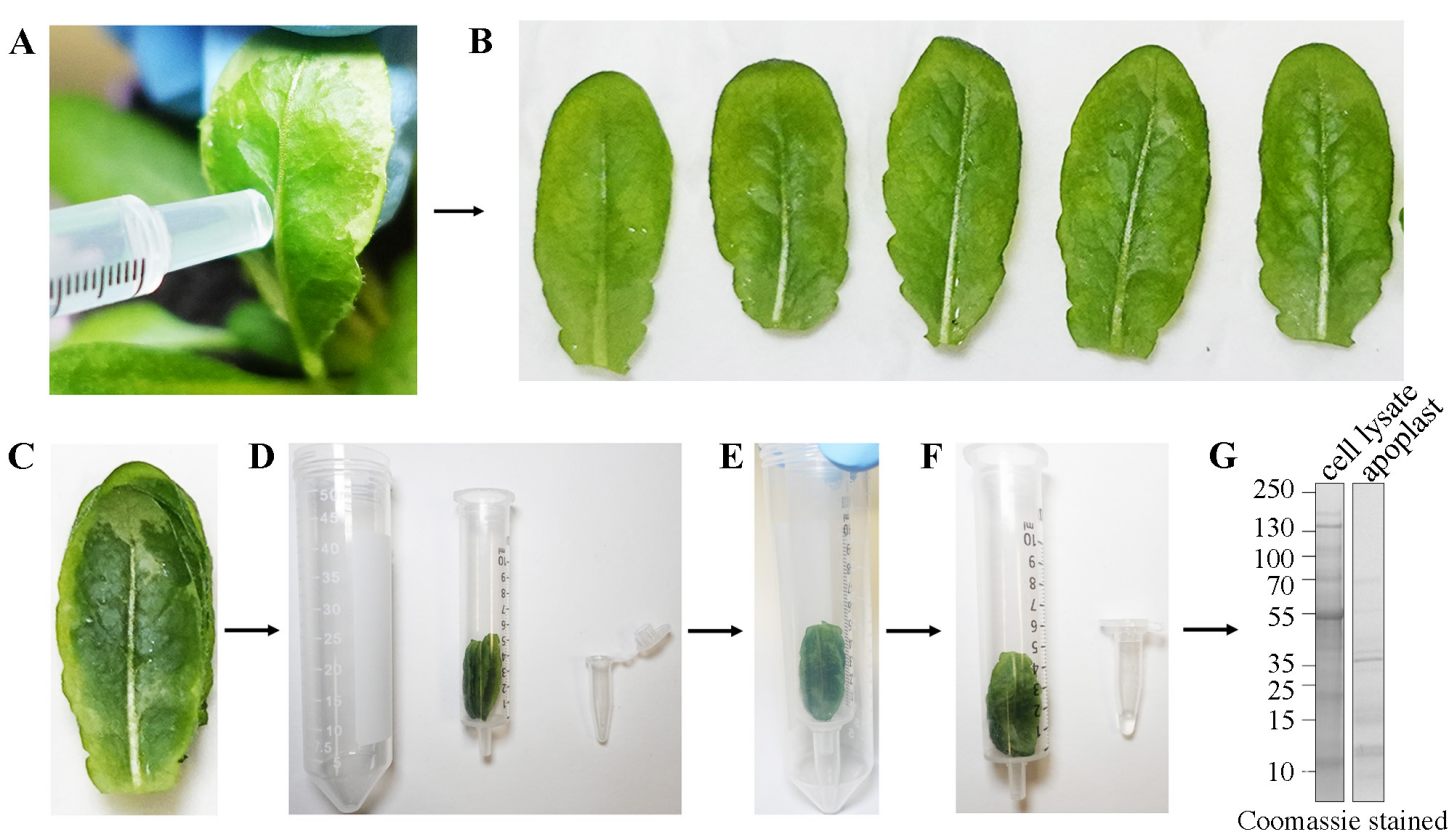
Apoplast flush collection protocol. (A) Leaf infiltration, (B) cutting, (C) stacking, (D, E) centrifugation assembly, and (F) centrifugation (see details in the procedure section). (G) Representative SDS-PAGE gel image showing total protein in cell lysate and apoplast flush using Coomassie stain.

1. Fill a 1 mL needleless syringe with the infiltration buffer (see Recipes).

2. Infiltrate the buffer in the leaf by gently pressing the syringe on the abaxial side (underside) while holding the adaxial side (upper side) with a fingertip (see Figure 2).

3. Observe the buffer spreads uniformly within the leaf slowly (hard pressing the syringe may damage leaf tissues, resulting in cytoplasmic protein contamination). Infiltrate the whole leaf.

4. Slice the leaves without the petiole and place them on a tissue paper to remove excess solution. Stack together 6–8 leaves, roll, and load into a 10 mL syringe with the tip facing down.

*Note: Minimum leaf cutting using a sharp blade is ideal to limit cytosol contamination from any damaged cells.*

5. Place a 0.5 mL microfuge tube on the bottom of a 50 mL Falcon tube and place the syringe containing leaves on the microfuge tube. Close the Falcon tube and centrifuge at 750× *g* for 4 min at 4 °C using a fixed-angle rotor.

*Note: Syringe flanges need to be removed by blade to fit in Falcon tubes; only the barrel is used.*

6. During centrifugation, apoplastic flush will be collected in the 0.5 mL microfuge tube. Carefully remove the assembly and collect the apoplastic flush (from 6 leaves, approximately 50–100 μL). The apoplastic flush generally looks colorless. A faint green color may indicate cell rupture and cytosol leak, making the sample unsuitable for analysis; please see General notes).

7. Determine protein concentration using Bradford assay. For mass spectrometry analysis, we used approximately 50 μL of sample at 1 μg/μL concentration (approximate protein concentration in apoplast extract expected is 0.5–2.5 μg/μL, although this may vary based on the experimental setup, for example, extraction efficiency, age of the plant, and treatments).

8. Add Laemmli buffer (1×) to the samples and incubate at 95 °C for 5 min.

**Pause point:** Apoplastic flush can be stored at -20 °C.

*Note: Protein in infiltration solution at -20 °C results in precipitation. If the Laemmli buffer is not used, we suggest adding a suitable buffer to ensure sample viability. Recommended buffer is 50 mM Tris, 150 mM NaCl, pH 7.8.*

9. Run samples on SDS-PAGE to check the quality.

*Note: The SDS-PAGE analysis of apoplastic flush shows no apparent band of Rubisco large chain (~53 kDa) compared to the total fraction, suggesting that the sample is suitable for MS analysis. If cytoplasmic contamination is higher than 1%, then repeat sample preparation using centrifugation at a lower speed. In case of low yield, we recommend increasing centrifugation time.*

**C. Protein digestion and peptide desalting**

*Notes:*

*1. The filter-aided sample preparation (FASP) protocol was adapted from Wisniewski et al. [22].*

*2. Samples must be reduced prior to starting the protocol using the reducing buffer and the recommended incubation protocol. The standard protocol recommended for Laemmli buffer is incubation at 95 °C for 5 min; for SDT-lysis buffer (see Recipes), incubate at room temperature for 30 min.*

1. Mix up to 50 μL of apoplastic flush in Laemmli buffer with 200 μL of UA (see Recipes) in a filter unit (a 30 kDa cutoff filter is used as standard; if needed, a filter with a lower cutoff may be used) and centrifuge at 14,000× *g* for 15 min using a fixed-angle rotor (additional volume will require repetition of this step).

2. Add 200 μL of UA to the filter unit and centrifuge at 14,000× *g* for 15 min.

3. Discard the flowthrough from the collection tube.

4. Add 100 μL of IAA solution (see Recipes) to the filter unit and mix at 600 rpm in a thermo-mixer for 1 min. Incubate without mixing for 20 min in the dark at room temperature.

5. Centrifuge the filter units at 14,000× *g* for 10 min at room temperature and discard the flowthrough.

6. Add 100 μL of UA to the filter units and centrifuge at 14,000× *g* for 10 min. Repeat this step twice.

7. Add 100 μL of 50 mM ammonium bicarbonate (see Recipes) to the filter units and centrifuge at 14,000× *g* for 10 min. Repeat this step twice.

8. Add 120 μL of 50 mM ammonium bicarbonate with Trypsin (enzyme to protein ratio 1:100) and mix at 600 rpm in a thermo-mixer for 1 min.

*Note: Take care to avoid introducing bubbles.*

9. Incubate the filter units at 37 °C for a minimum of 4 h to a maximum of overnight.

10. Transfer the filter units to new collection tubes and centrifuge the filter units at 14,000× *g* for 10 min.

11. Add 50 μL of 10% acetonitrile (see Recipes) and centrifuge the filter units at 14,000× *g* for 10 min. Discard the flowthrough and reuse the collection tube.

12. Add TFA to a 1% final concentration to acidify the sample and dry down in a vacuum centrifuge.

**Caution:** TFA is a skin sensitizer and can cause severe burns and eye damage. It should be dispensed in a fume hood on a tray so that any spillages are easier to contain/clean up.

13. Reconstitute the samples in 100 μL of 100 mM TEAB (see Recipes). Determine peptide concentration using a Nanodrop at a wavelength of 205 nm (the expected range of peptide concentration is 10–100 μg/ μL).

**Pause point:** Samples can be stored at -20 °C.

**D. TMT labeling of peptides**

*Note: Label peptides with TMT reagents (6-plex, 10-plex, 16-plex, 18-plex).*

1. Immediately before use, equilibrate the 6-plex TMT Label Reagents to room temperature. For the 0.8 mg vials, add 41 μL of anhydrous acetonitrile to each tube. For the 5 mg vials, add 256 μL of solvent to each tube. Allow the reagent to dissolve for 5 min with occasional vortex mixing. Briefly centrifuge the tube to gather the solution.

*Note: Reagents dissolved in anhydrous acetonitrile are stable for one week when stored at -20 °C. Anhydrous ethanol can be used as an alternative solvent, but it is not recommended for stock solution storage.*

2. Add 8 μL of the TMT label reagent to each 25 μL sample (final concentration of acetonitrile 24%).

*Note: Use 10–25 μg of peptide for each labeling reaction for optimal results.*

3. Incubate the labeling reaction for 1 h at room temperature.

**Critical:** To reduce the quantification errors caused by different labeling times, the incubation time for each channel should be measured precisely.

4. Quench the TMT-labeled reaction by adding 8 μL of 5% hydroxylamine to the sample and incubate for 15 min (final concentration of hydroxylamine 0.23%).

*Note: Do not quench the reaction until the QC run is complete.*

5. Combine TMT-labeled samples to a total of 3 μg on a 96-well plate. The remainder of the samples can be stored at -80 °C until the QC run has been performed.

6. Dry the sample using a Speedvac.

7. Resuspend the sample in 20 μL of sample resuspension buffer (see Recipes).

8. Proceed with this sample for mass spectrometry analysis.

**E. Liquid chromatography–mass spectrometry (LC–MS) analysis**

Analyze samples using tandem mass spectrometry (nLC-ESI-MS/MS) coupled to a HPLC system. The instrumental setup can be different due to the large variation in mass spectrometers and C18 chromatography systems. We analyzed TMT-labeled peptide samples on an Orbitrap Elite/Fusion MS coupled to a Dionex Ultimate 3000 RSLC nanoflow system. Ionization of LC eluent was performed by interfacing the LC coupling device to a NanoMate Triversa (Advion Bioscience) with an electrospray voltage of 1.7 kV. An injection volume of 5 μL of the reconstituted TMT-labeled samples were desalted and concentrated for 10 min on a trap column (0.3 × 5 mm) using a flow rate of 25 μL/min with 1% acetonitrile with 0.1% formic acid. Peptide separation was performed on a Pepmap C18 reverse-phase column using solvent A and solvent B (see Recipes) gradient at a fixed solvent flow rate of 0.3 mL/min for the analytical column. The solvent gradient was 4% B (reverse-phase UHPLC solvent B) for 1.5 min, 4%–60% for 178.5 min, 60%–99% for 15 min, held at 99% for 5 min (total run length 210 min). A further 10 min at initial conditions for column re-equilibration with 4% B was used before the next injection. The Orbitrap Elite/Fusion acquires a high-resolution precursor scan at 120,000 resolving power (over a mass range of m/z 375–1,500), followed by top speed (3 s) HCD (higher-energy collisional dissociation) fragmentation detection of the top precursor ions from the MS scan with Orbitrap detection of the TMT quantitation mass tag and peptide fragments at a resolution of 60,000 in MS2 spectra. HCD energy and isolation width was adjusted depending on the charge of the precursor ions: 2+ isolation width of 1.2 m/z with 40% HCD, 3+ isolation width of 0.7 m/z with 38% HCD, and 4–6+ isolation width of 0.5 m/z with 38% HCD. Precursors were excluded from fragmentation for the next 45 s.

**F. MS data analysis**

A variety of tools are available for MS data analysis, including Proteome Discoverer (PD), which utilizes “processing” and “consensus” workflows to process and report MS data collected from MS instruments. PD utilizes search engines, including Sequest HT, Mascot, MSPepSearch, and CHIMERY^TM^, for data processing. We used the MSPepSearch search engine node because it supports the use of spectral libraries, which makes the analysis process faster. Additionally, we observed a 30% improvement in the number of peptide/protein hits obtained using spectral libraries compared to conventional Sequest HT and Mascot searches. In addition, PD3.0 and 3.1 enable the generation of theoretical spectral libraries for both unlabeled and TMT-labeled peptides for any genome. We successfully tested *Arabidopsis* theoretical spectral libraries, including unlabeled, TMT 6-plex, and TMT-pro generated using PD3.0 and PD3.1 software.

Here, we describe a step-by-step workflow for the analysis of *Arabidopsis* secretory proteome dataset using Proteome Discoverer^TM^ 3.0 software with the MSPepSearch search engine node.

**G. Prediction of spectrum library**

**Figure 3.**
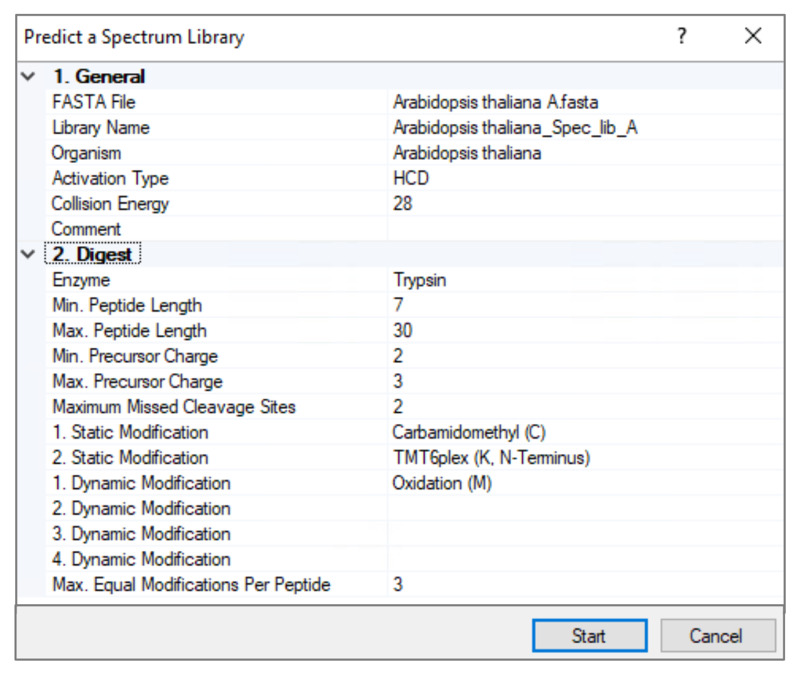
Prediction of TMT 6-plex labeled peptide spectrum library for *Arabidopsis* proteome using Proteome Discoverer^TM^ 3.0

*Note: Proteome Discoverer^TM^ 3.0 predicts spectral libraries with TMT 6-plex labeled peptides, whereas Discoverer^TM^ 3.1 predicts spectral libraries with TMT 6-plex as well as TMT pro (18-plex) labeled peptides. This protocol describes TMT 6-plex analysis using Proteome Discoverer^TM^ 3.0 (see Figure 3).*

1. Download *Arabidopsis* proteome from UniProt (ID3702) in a FASTA format.

**Critical:** The predicted spectral libraries are too large (32–34 GB), limiting PD3.0 performance. Therefore, we divided the *Arabidopsis* proteome into four different FASTA files (named A, B, C, and D).

2. Open Proteome Discoverer^TM^ 3.0 and select the FASTA file on the *Administration* start page.

3. Add all four *Arabidopsis* proteome FASTA files (one at a time).

4. Open *Spectral libraries* and click *Predict*.

5. Select the FASTA file.

6. Choose a name for the spectral library and organism.

7. Specify the parameters as follows: activation type, HCD; collision energy value, 28; enzyme, trypsin; minimum peptide length, 7; maximum peptide length, 30; min precursor charge, 2; max precursor charge, 3; maximum missed cleavage sites, 2; minimum mass of the precursor ion, 2.

8. Specify the static modifications as follows: Carbamidomethyl (C), TMT6-plex (K, N-terminus); specify dynamic modification, oxidation (M); specify the maximum number of times that the same dynamic modification can be used in a single peptide, 3.

9. Predict all four *Arabidopsis* spectral libraries.

**Optional:** Use our *Arabidopsis* spectral files (available upon request).

**H. Data analysis**

**Figure 4.**
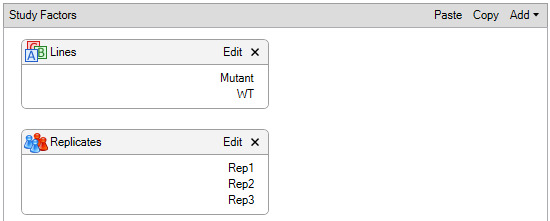
Design of study factors for the TMT-based quantitative proteomic analysis of the *Arabidopsis* proteome. The study factors show parameters, such as categorical factors, denoted as lines (upper box), and replicates (lower box), to create sample groups defined in the experiment.

1. In PD3.0, select *New Study/Analysis* on the start page.

2. Choose a name for the study, the root directory, the processing workflow, and the consensus workflow. Click *OK*.

3. In the *Study Definition* tab, add the quantification method: TMTe 6plex.

4. Click *Add Files* and select the mass spectrometry raw files.

5. In the *Study Definition* tab, select the study factors (biological replicates, numerical, and categorical) and set the parameters (see Figure 4).

*Note: We used replicate and categorical factors.*

**Figure 5.**
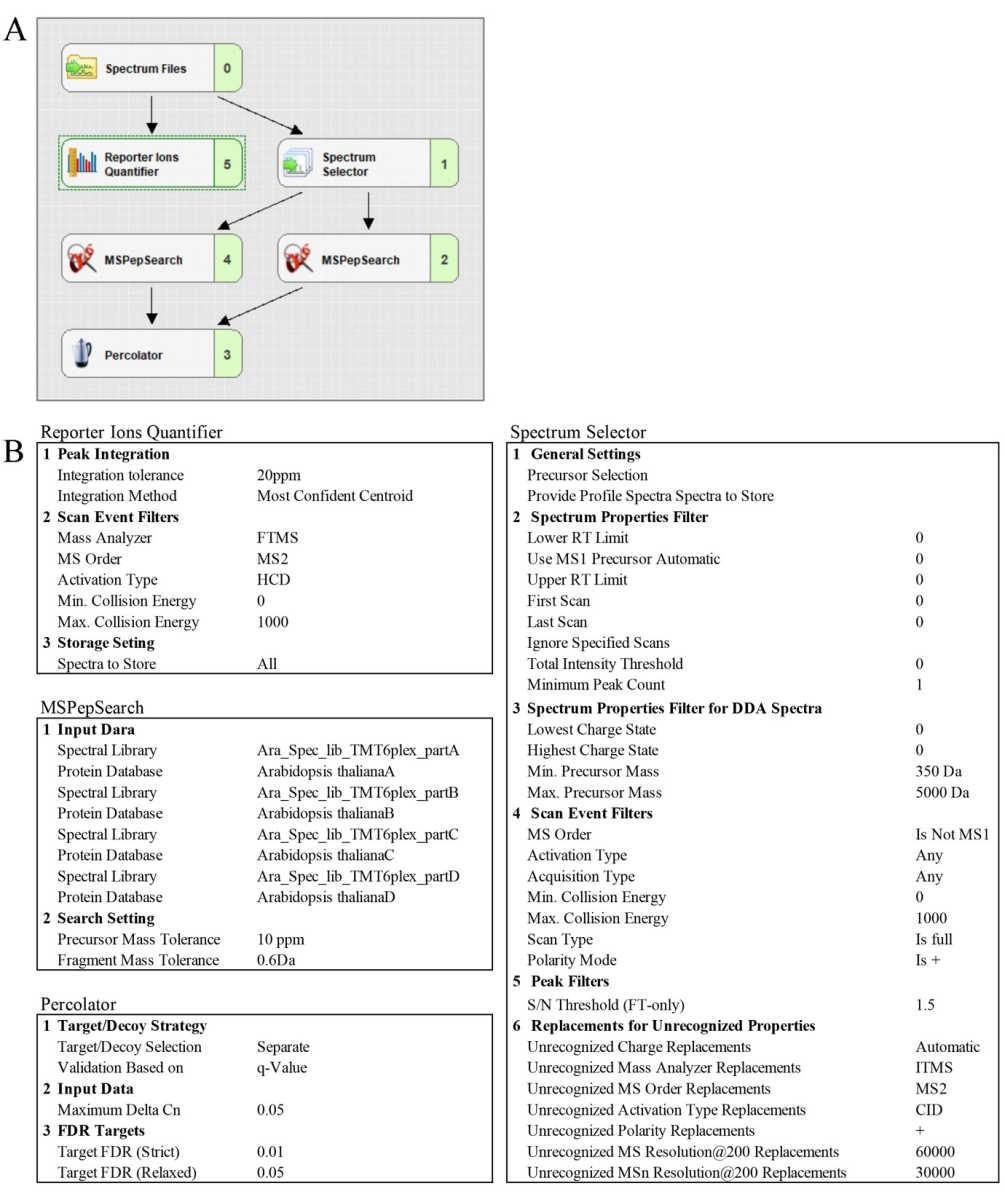
Proteome Discoverer setting for the TMT-based quantitative proteomic analysis of the *Arabidopsis* proteome. (A) Processing workflow. (B) Parameters for *Report Ion Quantifier, Spectrum Selector, MSPepSearch*, and *Percolator* nodes.

**Figure 6.**
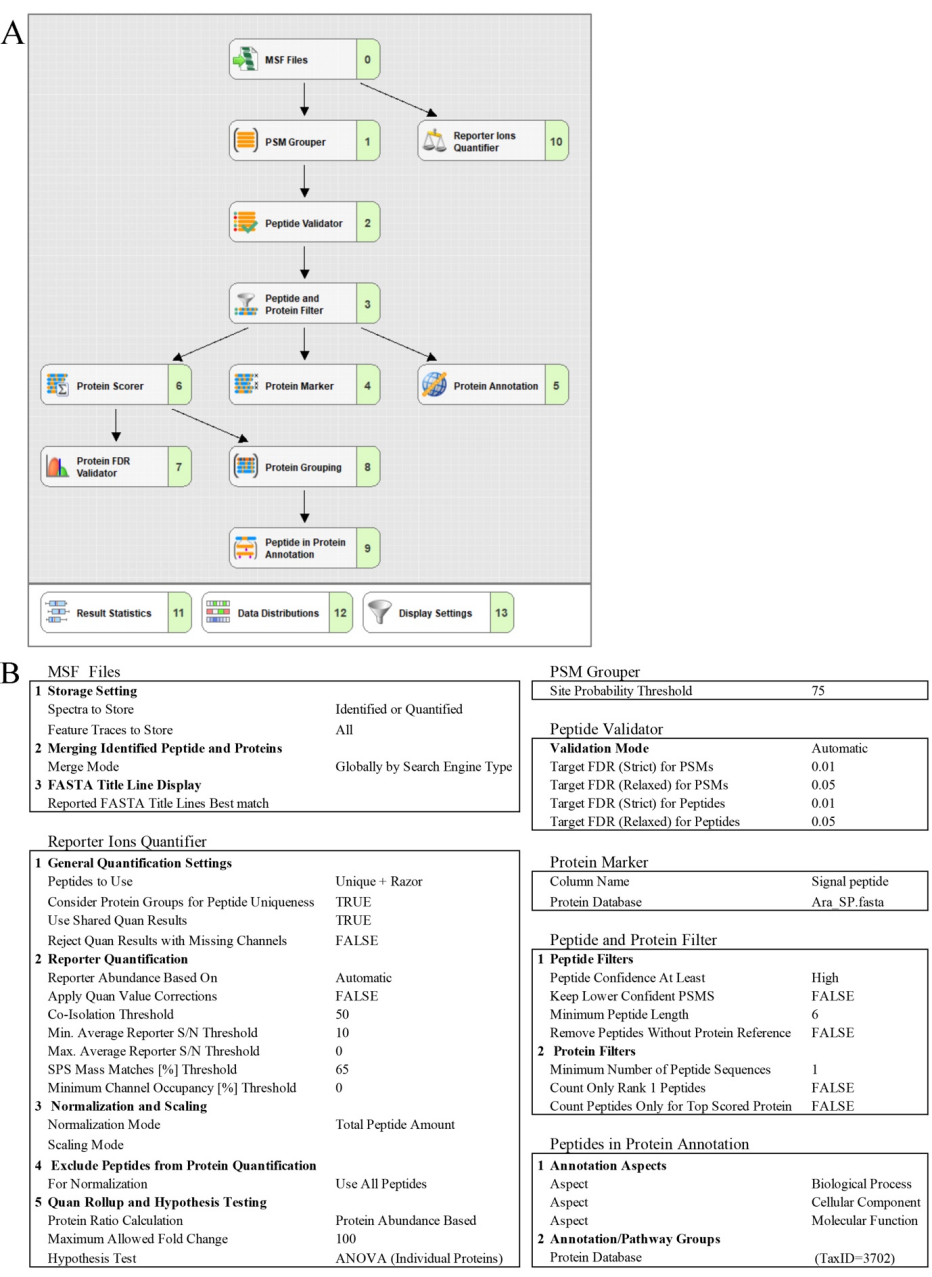
Proteome Discoverer setting for the TMT-based quantitative proteomic analysis of the *Arabidopsis* proteome. (A) Consensus workflow. (B) Parameters for *MSF Files, Report Ions Quantifier, PSM Grouper, Peptide Validator, Protein Marker, Peptide and Protein Filter*, and *Peptides in Protein Annotation*.

6. On the *Sample* tab, specify the sample type as *Control* and/or *Sample*.

7. Open *New Analysis* and drag the sample file to the *Analysis* section.

8. Create the processing (see Figure 5A, B) and consensus workflow (see Figure 6A, B) in the *Workflows* tab by dragging and connecting nodes from the *Workflow Nodes* to the *Workflow Tree*.

**Optional:** Use our workflow.

*Note: In the MSPepSearch node, select all four* Arabidopsis *spectral libraries (A, B, C, and D).*

**Figure 7.**
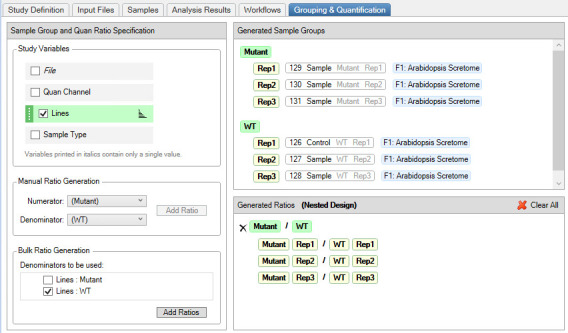
Proteome Discoverer setting for the grouping and quantification of *Arabidopsis* TMT-labeled peptides. Sample groups are generated by selecting the categorical factor (Lines), and mutants are ratioed against the WT to generate replicates in a nested design.

9. In the *Grouping and Quantification* tab, generate the *Sample Groups* by clicking the *Study Variables (Files, Quan Channels, Lines*, and *Sample Type*). In the example below, we used *Lines* to generate mutant/WT ratios with a nested design (see Figure 7).

10. For the identification of secretory proteins in the conventional secretory pathway, download the *Arabidopsis* protein database with signal peptides, transmembrane domains, and ER retention signals from UniProt in FASTA format. Add the FASTA files in PD 3.0 by selecting *FASTA files* on the *Administration* start page. Use these files in the *Protein Marker* node to annotate the secretory proteins.

In our previous studies, we classified a secretory protein that contains a signal peptide and lacks a membrane anchor and ER retention signal [4].

## General notes and troubleshooting

During the apoplast flush extraction from *Arabidopsis* leaves, caution is advised to minimize cell breakage and leakage throughout the process. Because intracellular protein leaking into the apoplast is inevitable, it is recommended that the contaminant background be below 1% and remain uniform across all samples.

To determine cytoplasmic contamination in the apoplast sample before mass spectrometry analysis, Rubisco can be detected by immunoblot analysis with anti-Rubisco antibodies, as described in Waghmare et al. [21]. If anti-Rubisco antibodies are not available, other cytoplasmic contaminants, such as malate dehydrogenase (MDH), 3 glucose-6-phosphate dehydrogenase (G6PDH), and glucose-6-phosphate (G-6-P) can be detected using commercially available assay kits [23].

In proteomics analysis, detection of Rubisco is unavoidable, as it is a highly abundant protein in plants and thus detectable even in highly pure apoplast samples. We recommend using different cytoplasm resident proteins as a measure of purity, including MALATE DEHYDROGENASE 1 (MDH, AT1G04410) and CATALASE 3 (CAT3, AT1G20620) as described before [23].

We recommend using at least two different approaches to evaluate apoplast sample purity. We suggest conducting a small aliquot of label-free mass spectrometry (see Sections C and E) to assess peptide preparation quality, considering budgetary constraints.

## Validation of protocol

This protocol (or parts of it) were used and validated in following research articles:

• Waghmare et al. [21]. SYNTAXIN OF PLANTS 132 underpins secretion of cargoes associated with salicylic acid signalling and pathogen defense. *Plant Physiology* (Figures 1E–G, 2A–E, and 3A show data from TMT–MS analysis of *Arabidopsis* leaf apoplast secretome).

• Baena et al. [6]. SNARE SYP132 mediates divergent traffic of plasma membrane H^+^-ATPase AHA1 and antimicrobial PR1 during bacterial pathogenesis. *Plant Physiology* (Figure 4A–C shows apoplast flush extraction and analysis of antimicrobial secreted cargo using immunoblot).

Apoplast purity was determined by immunoblotting analysis of Rubisco heavy chain protein (∼53 kDa), a highly abundant cytoplasmic protein, compared with total leaf tissue lysate (estimated >99%).

TMT labeling efficiency ranged between 97% and 99% across all samples (>95% acceptable). The robustness and reproducibility of the method were validated through variability in experimental replicates using hierarchical clustering and principal component analysis (PCA). The abundance patterns of secretory cargoes in the controls and treatments/mutants were consistent within the sample groups, indicating alterations in protein abundances only between the two groups. TMT–MS quantification of secretory proteins in the mutants/treatments was verified by immunoblotting analysis targeting PR2 protein (∼34 kDa), a secretory cargo that responds to pathogen treatment in our experiment. The results from immunoblotting analysis agreed with the TMT–MS analysis.
